# Supplementary material for: Are carbon emissions trading and green financial instruments synergistic? -Comprehensive quantitative research based on content analysis
Source: PLoS One. 2024 Mar 7;19(3):e0298601. doi: 10.1371/journal.pone.0298601 (PMC10919601; doi:10.1371/journal.pone.0298601)
Supplement: S1 Data — (ZIP) [file pone.0298601.s001.zip › Data/地级市Z_F工作报告.zip!/╔┬╬≈/╔╠┬σ╩╨/└┤╫╘╣1⁄2╓┌║┼í╛┬φ┐╦╩2╛▌═°í┐_2015.docx]

各位代表：

现在，我代表市人民政府向大会作工作报告，请予审议，并请市政协委员和其他列席人员提出意见。

一、市三届人大四次会议确定任务完成情况

2014年，面对复杂严峻的经济形势，在省委、省政府和市委的正确领导下，我们牢牢把握稳中求进、改革创新、提质增效的工作总基调，认真贯彻中省稳增长、促改革、调结构、惠民生、防风险各项政策，低调务实不张扬、埋头苦干，经过全市上下的共同努力，解决了一批难事，推进了一批大事，办好了一批实事，全市经济在上升通道的合理区间稳健运行。完成生产总值576.27亿元、增长11%，位居全省第四；财政总收入42.17亿元、增长6.2%，其中地方财政收入29.04亿元、增长13.1%；预计城乡居民收入分别为24930元、7094元，分别增长12%、14%；城镇登记失业率3.22%，物价上涨1.7%，明显低于控制指标。

(一)紧扣稳增长的关键环节精准发力，经济继续保持平稳较快增长。一是狠抓重点项目建设。160个市级重点项目全部开工建设，完成全社会固定资产投资625.16亿元、增长26%，位居全省第三，其中民间投资占到51%。丹江流域综合治理、西合铁路复线等重大项目顺利推进，“五件大事”取得积极进展，“一体两翼”中心城市建设开局良好，商丹园区招商引资和产业项目建设取得重大突破，西武客专商洛段预可研编制完成并上报中铁总公司，镇安抽水蓄能电站核准和电网接入方案上报国家待批，山柞高速公路项目前期工作全面完成。二是力促工业达产达效。出台了一系列稳产促销保市场综合举措，规模以上工业总产值632.47亿元、增长29.5%，增加值171.09亿元、增长20%，均位居全省第一，成为稳增长的中坚力量。三是着力扩大城乡消费。社会消费品零售总额136.87亿元、增长12.4%，对经济增长的贡献进一步提高。四是大力发展现代农业。在全省率先启动秦岭生态农业示范市创建，积极推进“一村一品”，创建省级现代农业园区17个，发展市级龙头企业20个，建设基本农田9.15万亩，粮食总产达到52.05万吨，农业发展的基础更加稳固。

(二)围绕打造商洛经济升级版优化结构，转型发展与提质增效迈出较大步伐。充分发挥产业园区的聚集带动作用，加快推进现代材料、现代中药、绿色食品等主导产业转型升级，大力培育新能源、电子信息、智能制造等新兴产业，光伏农业、高端饮用水、工业“机器人”、药物中间体提取等33个产业项目建成投产，商丹园区电动客车、山阳中德产业园汽车液压缸等重大战略项目启动建设。积极推进文化产业与旅游产业融合发展，商於古道棣花景区建成开园，我市创建为“中国文化经济活力城市”。成功举办了2014中国秦岭生态旅游节和首届中国环秦岭公路自行车赛，金丝峡5A级创建通过国家旅游局资源评估，柞水溶洞创建为国家4A级景区，创建3A级景区2个，创建验收“美丽乡村”33个，全市接待游客3005.65万人次，综合收入153.29亿元。大力推进科技创新和技术进步，技术合同成交额6977万元，专利授权160件，分别增长60%、16.7%，科技对经济增长的贡献率提高3个百分点。

(三)坚持以人为核心推进新型城镇化，统筹城乡发展取得显著成效。出台了加快“一体两翼”中心城市建设的决定，启动实施6大类65个重大建设项目，完成投资32.7亿元，其中47个项目建成。市区“八大工程”建设进展顺利，环城北路、商鞅大道西段、市一级客运中心及西街旧城改造安置房等工程主体基本完工，商州至丹凤、商州至洛南两条城市交通干线全面建设，国家园林城市创建、智慧城市建设等工程加快推进。投资27.31亿元用于县城、重点镇和文化旅游名镇建设，公路交通等城乡基础设施持续改善，改造干线公路55公里、县乡公路280公里，新修通村水泥路187公里。完成电网建设投资3.26亿元，建成通信基站1146个。把陕南移民搬迁、保障性住房建设和新型城乡社区建设有机结合，建设陕南移民搬迁集中安置点112个，实施搬迁1.6万户、6.08万人，落实国开行贷款35.6亿元用于棚户区改造，新建保障性住房1.52万套，建成新型城乡社区75个，7.5万农村居民进城落户，全市城镇化率达到47.5%。

(四)针对突出问题实施铁腕治污，生态环境建设继续向纵深推进。创建国家循环经济示范城市顺利推进，建成循环示范城市创建项目80个、国家尾矿资源综合利用示范基地项目62个。治污降霾·保卫蓝天行动成效明显，淘汰黄标车及老旧车辆5365辆，拆改燃煤锅炉31台，空气质量位居全省首位。强力推进丹江等流域污染防治，实施污染防治项目102个，丹江出境断面水质稳定达标，为“一江清水供京津”作出重大贡献。狠抓节能减排和过剩产能化解，实施节能减排项目43个，淘汰过剩产能2.7万吨，节约标煤4.98万吨，单位GDP能耗下降3%，二氧化硫、化学需氧量、氨氮和氮氧化物排放分别削减0.3%、1.8%、2.7%和5%。大力实施生态建养和农村清洁工程，加快推进小流域综合治理，造林绿化32.55万亩，治理水土流失702平方公里，我市在全省率先入选“全国生态文明示范工程试点市”。

(五)按照兜底补短要求办好民生实事，改革发展成果不断惠及人民群众。在全市开展了六项民生政策大检查，有效解决了政策落实不到位、部分群体保障标准低、兜底政策不完善等问题。扎实开展“双包双促”精准扶贫，结对帮扶贫困户7.4万户，9.3万贫困群众实现脱贫，解决安全饮水13.13万人。就业工作创近年来最好水平，全市新增城镇就业1.83万人，大学生初次就业率90.56%，转移农村劳动力60.4万人。社会保障水平不断提升，建立健全了城乡居民养老保险丧葬补助金制度和城镇居民大病医疗保险二次报销制度，城镇职工基本养老保险待遇实现十连增，城乡居民养老保险待遇提升10%，医疗、失业、工伤、生育等保险水平持续提高。社会救助范围更加广泛，下拨各类社会救助资金6.82亿元，实施医疗救助11.27万人次，临时救助9200户次。社会养老服务体系不断完善，新增养老床位2000张，建成残疾人托养及康复中心21个。

(六)坚持共建共享提升公共服务，各项社会事业全面协调发展。一是各类教育事业均衡发展。山阳、商南通过国家义务教育发展基本均衡县评估认定，商南“教育强县”、丹凤“双高双普”创建通过省政府命名表彰，全市高考二本以上上线率居全省第一。完成了市直职教资源整合工作，新、改扩建公办幼儿园39所，营养改善计划实现全覆盖，惠及14.2万名中小学生。二是卫生服务能力有效提升。县乡医疗机构定向招聘医学毕业生158名，大病保障病种达到24种。计生特殊困难家庭扶助金补助标准实现城乡统一，“单独两孩”政策平稳实施，人口自增率为3.17‰。三是公共文化服务体系不断完善。完成广播电视户户通20.35万户，“三馆一站”全部免费开放，我市创作剧目《带灯》、《沉香》在西北五省区秦腔艺术节和第七届陕西省艺术节上荣获多项大奖，创作的花鼓历史剧《聂涛》荣获陕西省第十三届精神文明建设“五个一”工程优秀作品奖。竞技体育和全民健身运动深入开展，我市在十五届省运会上获得了14金7银7铜的好成绩。四是平安商洛建设持续加强。食品药品安全监管力度加大，全市安全生产形势持续稳定，各类安全事故起数下降14.5%。积极推进社会稳定风险评估，全面排查化解社会矛盾，社会治安环境进一步改善，公众安全感和满意度连续七年居全省前列。

(七)依靠改革破解体制机制障碍，发展的内在活力和内生动力加快释放。坚持以问题为导向全面深化行政、经济、生态、农村、文化、社会等领域的改革。启动了新一轮市、区县政府机构改革和镇村综合改革，全市镇、村撤并率分别达到22.2%、34.1%。取消和下放市级行政审批事项93项、精简幅度25.3%。全面实行先照后证工商登记制度，清理规范涉企行政收费24项。财税体制和投融资体制改革稳步实施，国资国企等五项改革进展顺利。行政管理体制和经济体制改革不断深化，有效地激发了市场主体活力，新增各类市场主体1.3万户，其中企业1735户、个体工商户1.1万户。非公有制经济占比达到51.58%，位居全省第三。招商引资到位资金365亿元、增长17.8%，外贸进出口总额3.62亿美元、增长5.76倍。金融机构新增存贷款余额82.08亿元、37.04亿元，分别增长13.93%、14.93%。

(八)结合群众路线教育实践活动狠抓自身建设，政府工作效能进一步提升。群众路线教育实践活动整改措施有效落实，及时解决了一批群众反映的突出问题。预决算和“三公”经费支出公开扩大到所有区县，全市压缩“三公”经费开支3771万元、同比下降23.2%，“文山会海”得到有效遏制，清理超编超配公务用车81台，停建楼堂馆所项目13个。市区县政务服务中心标准化建设加快推进，县镇村三级群众办事干部代办制度全面实行，新招聘社区工作人员54名。深入开展“政府执行力提升年”活动，实行重点工作月报告制度，确保了政令畅通和工作落实。把依法行政作为政府工作的生命线，自觉接受市人大的法律监督和市政协的民主监督，办理人大代表建议184件、政协提案289件。

同时，国防动员、民族、宗教、外事、侨务、对台、地震、气象、测绘、档案、统计、人防、水文、方志、慈善、供销、无线电、工商联、妇女儿童、红十字会等领域的工作也都取得新的成绩。

各位代表，过去的一年，面对贯穿全年的经济下行压力，我们科学应对，保持了经济平稳较快增长；面对50年不遇的特大旱灾，我们奋力救灾，最大限度地减少了灾害损失。这些成绩的取得来之不易，是省委、省政府和市委坚强领导的结果，是市人大、市政协监督支持的结果，是全市上下团结奋斗的结果。在此，我谨代表市人民政府，向全市人民、驻商部队指战员、武警官兵和公安民警致以崇高的敬意！向大力支持政府工作的各位代表、各位委员和社会各界人士表示衷心的感谢！

在看到成绩的同时，我们也清醒地看到，由于受多种因素影响，去年部分指标完成与预期目标有差距，少数重点项目建设没有达到计划进度。我市发展仍面临着许多突出问题和深层次矛盾，主要是：经济发展的资源环境约束加剧，污染治理和结构调整任务艰巨，科技创新动力和市场主体活力仍显不足，城镇化和城乡居民收入水平低于全省，市场监管、社会管理和公共服务能力有待加强，法治政府建设和作风建设仍是长期任务。对此，我们要高度重视，努力加以解决。

二、新常态下我市发展的基本要求和今年主要预期目标

当前，宏观经济环境正在发生深刻变化，中央作出了我国经济进入新常态的战略判断，保持中高速增长、迈向中高端水平，既是新常态的发展必然，也是今后工作的基本遵循。

我们要正确认识新常态。速度变化、结构优化、动力切换的新常态，既是严峻挑战，更是难得机遇。我们必须承受经济增速连续波动下滑的压力，资源依赖和投资拉动粗放发展面临的结构调整压力，土地、资金和人才等要素的保障压力，财政收入增长放缓与民生刚性支出持续增加的压力，长期累积形成的地方性债务短期难以消化的压力。但是我们有信心在市委的坚强领导下，变压力为动力、化挑战为机遇。信心在于，国家实施“一带一路”、创新驱动和优先推进西部大开发战略，加快实施关天经济区、丹江口库区及上游地区发展等一系列重大规划，省委、省政府加快丝绸之路经济带新起点建设，对我市来说是重大历史机遇；信心在于，国家加快中西部铁路建设、推进棚户区改造提速、加大重大水利工程投资，我市将获得更多的支持；信心在于，近年来我们不断深化市情认识，进一步理清了发展思路，一批重大项目积极推进，战略新兴产业快速兴起，为长远发展聚集了正能量；信心在于，在全国各地共同遭遇“三期叠加”阵痛背景下，我市的生态、资源等优势更加凸显，蕴含着巨大的发展空间与潜力，必将在新一轮竞争中赢得先机；信心在于，全市广大干部群众的思想不断解放、作风持续转变，人心思变、人心思进、人心思干的氛围日益浓厚，我们完全有条件有能力保持经济平稳较快发展。

我们要主动适应新常态。要系统谋划促转型，发挥优势补短板，开拓创新破难题，努力把自己的事情办好。要全面深化改革，进一步释放改革红利；要培育新兴产业，构建具有商洛特色的现代产业体系；要持续扩大开放，不断催生新的经济增长点；要严守生态红线，全面推进循环发展、绿色发展、低碳发展；要强化创新驱动，最大限度地激发内生增长动力；要持续改善民生，努力提高全市人民的幸福指数。

今年政府工作的总体要求是：全面贯彻落实党的十八大和十八届三中、四中全会精神，按照省委十二届六次全会和市委三届七次全会的部署，以保持经济运行在合理区间、提高发展质量和效益为中心，牢牢把握“稳中求进、改革创新、提质增效”的工作总基调，紧紧围绕“依托大西安、建设大商洛、打造大品牌”的工作思路，继续打造“两大板块”、做强“两大经济”、实施“两大工程”，统筹推进转方式、调结构、促改革、惠民生、防风险等各项工作。

主要预期目标是：生产总值增长11.5%左右，财政总收入、地方财政收入同口径分别增长10%和12%左右，城乡居民收入分别增长11%和13%左右，城镇登记失业率控制在3.9%以内，CPI控制在3%左右，节能减碳及四项减排指标完成省上下达任务。需要说明的是，在经济发展的新常态下，确定11.5%左右的增速，是一个预期性、区间性、引领性目标，是贯彻省委、省政府要求并结合商洛实际作出的安排，既是促就业、保民生的刚性需求，也是全面建成小康社会的目标追求，更是凝聚发展信心的现实需要，兼顾了需要与可能、当前与长远。我们一定要认清形势、保持定力，抢抓机遇、主动作为，努力创造商洛发展的新成就。

三、2015年的主要工作任务

(一)以稳增长为目标保持经济运行在合理区间

充分发挥投资对稳增长的关键作用，以重点项目建设进度和整体质量保经济增长速度与效益。通过扩大招商引资、降低准入门槛、推广PPP模式、强化要素保障、落实项目质量评估和目标考核制度等综合措施，实施市级重点项目160个，全社会固定资产投资增长20%以上。重点抓好电动客车、比亚迪锂离子电池电解液、中德产业园汽车液压缸、硅科技产业园、秦岭“飞行小镇”等重大产业项目，力促奥科纳米级材料产业园、欧珂医药产业扩能、必康医药产业园二期等项目建成投产。全力促进西合铁路复线建成投运，加快市妇幼保健院、市精神病院、全科医师培训基地、县级中医院等公益设施项目建设。继续狠抓“五件大事”不放松，争取镇安抽水蓄能电站、山柞高速等重大项目早获批、早开工。紧盯国家政策取向，编制“十三五”规划，策划实施一批重大战略项目。通过重大项目建设，拉动当期增长，增添发展后劲。

着力增加城乡居民收入和培育消费新业态，千方百计激活消费需求。一是完善城乡居民收入增长联动机制。积极推进收入分配制度改革，稳步推进工资制度改革，适时提高最低工资标准，建立县以下机关公务员职务与职级并行、职级与工资挂钩制度。完善工资集体协商制度，强化企业工资指导线作用，健全农民工工资支付保障机制，深入实施农民收入倍增计划。二是调动市场力量培育消费新业态。瞄准群众多样化需求，大力培育休闲、健康、养老等新兴消费，积极引进电商、物流龙头企业，创新发展互联网内容产业和衍生产业，促进电子商务和信息消费快速发展。实行公租房与廉租房、经济适用房与限价商品房并轨，出台收购商品房补充保障房政策，放宽公积金提取条件，引导房地产业健康发展，稳定群众住房消费。三是努力营造良好的消费环境。落实职工带薪休假制度，健全完善社会信用体系，严厉打击制假售假、商业欺诈等违法行为，努力保持价格总水平基本稳定，让人民群众安全、放心消费，社会消费品零售总额增长13%。

坚持“引进来、走出去”相结合，统筹推进招商引资和外向型经济发展。一是狠抓投资环境建设。抓住陕西建设丝绸之路经济带新起点的机遇，放宽商贸流通、电子商务、教育医疗等领域的投资准入限制，打造“暖人心、留住人、有发展”的投资环境。二是深入开展招商引资。建立赴京津冀、长三角、珠三角等发达地区常态化招商机制，大力开展节会招商、专题招商、叩门招商，加强重大签约项目跟踪落实，积极引进新的战略合作伙伴。三是实施外贸孵化器工程。依托商山物流园建立二类陆路货运口岸，争取独立的报关、商检权。继续抓好商南中小企业孵化园等省级出口基地建设，以光伏产品、电子产品、生物医药、农特产品出口为重点，培育壮大一批骨干出口企业，确保外贸进出口稳定增长。

(二)以迈向中高端水平为导向促进结构调整和产业升级

支持优势骨干企业稳产促销与培育新兴产业同步发力，加快打造商洛经济升级版。一是支持企业稳产促销。建立“政银企”季例会制度，加强生产企业运行保障，推行企业互保联保和融资租赁，支持企业上市、发行债券和票据，力促骨干企业扩大产能、开拓市场，主要工业品产销率保持在90%以上，规模以上工业增加值增长18%。二是扶持壮大主导产业。围绕做大做强现代材料、现代中药、绿色食品等主导产业，大力发展市场调研、技术研发、品牌设计等工业服务业，加快发展物流分销、品牌营销、售后服务等市场服务业，实现产业链条向两端延伸、规模和效益持续提升。三是培育发展新兴产业。突出抓好光伏农业及分布式光伏发电等项目建设，大力发展太阳能、风能等新能源产业，培育壮大智能制造等新兴产业，加快高端饮用水产业规模化发展。四是狠抓工业园区建设。支持商丹园区建成省级高新技术开发区，全力推进洛河市级工业园区基础设施建设，加快13个县域工业集中区扩容提质，实现更多企业入驻和产业项目建成投产。

依托特色农业园区加快转变农业发展方式，进一步提高农业生产的综合效益。一是抓好特色农业园区建设。鼓励农村土地经营权规范有序流转，大力发展多种形式的适度规模经营，新建市级以上特色农业园区10个。二是做大农业特色优势产业。以创建秦岭生态农业示范市为抓手，将产业培育、品牌打造相结合，大力发展核桃、板栗、山羊、生猪、土鸡、蔬菜、香菇、木耳等商洛特色农业“八大件”。强化“米袋子”、“菜篮子”行政首长负责制，粮食总产稳定在55万吨左右，新发展蔬菜2万亩。三是健全现代农业服务体系。加快培育新型农业经营主体，扶持龙头企业和专业社发展，完善农产品质量安全追溯和食品安全考核评价制度，继续做好农产品“三品一标”认证。四是持续改善农业基本生产条件。加快人工影响天气基地建设，狠抓农田水利和防洪工程建设，新修改造基本农田5万亩，恢复新增灌溉面积2万亩。策划实施一批水源水库项目，解决6万人的饮水安全问题。五是坚决落实强农惠农富农政策。建立健全三农投入稳定增长机制，加快农村土地确权颁证进度，探索建立农村产权交易市场。

大力发展旅游、文化、休闲等产业，全面提升服务业规模和层次。一是加快大型景区提档升级。全面实施《大秦岭生态旅游核心区规划》，突出抓好蟒岭郊野公园、江山风景区、营盘旅游产业园、洛河源旅游度假区建设，继续抓好金丝峡、牛背梁、天竺山等3个5A级景区创建，全力推进丹江漂流、老君山、塔云山、月亮洞及棣花文化景区等5个4A级景区创建。二是精心筹办“一节一赛”。加强与国内外知名旅游电商合作，精心筹办2015中国秦岭生态旅游节，高标准举办2015中国环秦岭公路自行车赛，力争全年接待游客突破3300万人次。三是积极培育特色文化产业。全力推进商於古道文化景区建设，打造我市文化旅游精品带、新型城镇化建设示范带和新的经济增长带，推动文化产业更好更快地发展，力争文化产业增加值增长18%以上。四是大力发展休闲健康养老产业。支持民间资本投资养老服务，鼓励社区医疗、养老机构融合发展。支持民俗体验、观光农业、农家乐、自驾游等特色休闲产业加快发展，打造“秦岭原乡”休闲旅游示范区。

围绕结构调整实施创新驱动战略，着力构建以企业为主体的创新体系。一是继续深化“校(院)企”合作。支持企业建立重点实验室、工程技术中心等研发平台，推动市内企业与省内外高校、科研院所、中介组织协同创新，全力推进先进制造、新材料、现代农业、节能环保等领域关键技术应用，促进科技成果尽快就地转化。二是切实优化科技创新环境。支持金融机构发展科技金融业务，探索科技贷款风险补偿和知识产权质押融资模式。认真落实引进优秀专业人才暂行办法，采取股权激励、销售收入提成、成果处置收益等分配方式，激发广大科技人才的创新动力和创业激情。三是推进金融和保险服务创新。稳步推动融资性担保体系建设，鼓励小额贷款和融资担保公司增资扩股，完成山阳、丹凤两县农村信用社改制任务，支持长安银行设立小微支行，推进农行、邮储等金融机构业务向镇村延伸，全年新增贷款40亿元以上。扩大政策性保险业务，完成保费收入11亿元。

(三)以深化改革为动力促使发展红利充分释放

加快推进经济体制改革，进一步激发市场主体活力。一是完善非公有制经济发展机制。积极推进商事制度改革，进一步放宽市场主体登记条件，加大实体经济扶持力度，加快促进中小企业和小微企业发展。二是加快推进国资国企改革。大力发展混合所有制经济，支持城投、交投、商丹园区建设开发、中小企业担保等市属国有企业发展，组建商洛文化旅游投资集团公司，实施国有企业经营体制和负责人薪酬制度改革。三是积极推进财税体制改革。认真执行新《预算法》，全面加强预算约束，规范政府债务管理，稳步推进“营改增”，通过整合专项资金、实施“拨改投”等方式创新财政投入机制，促进财税、金融和产业政策有效衔接。

继续深化行政管理体制改革，简政放权提高政府效能。一是深入推进政府机构改革。完成市、区县政府机构改革和镇村综合改革任务，分类推进事业单位改革，开展法人治理结构改革试点。二是进一步推进审批制度改革。继续精简市级行政许可审批事项，全面清理非行政许可审批事项，实行保留审批和收费目录清单管理。三是加快改革试验区建设。认真落实加快商丹园区发展的政策措施，探索南秦新区建设的体制机制和开发模式，建立洛河市级工业园区市县共建体制机制，健全完善土地和财税分享机制，积极发展“飞地经济”，全力加快园区(新区)发展步伐。

稳步推进社会领域各项改革，加强政府治理能力建设。一是深化社会组织管理改革。加强监管和第三方评估工作，鼓励和支持社会力量参与社会治理。建立统一规范的社区工作标准体系和评价制度，做好第九次村委会换届工作，充分发挥基层自治组织的功能。二是完善社会矛盾化解机制。全面推进社会稳定风险评估工作，完善人民调解与行政调解、司法调解联动工作体系，落实涉法涉诉信访依法终结制度，有效预防和化解社会矛盾。三是全面加强安全生产管理。严格落实安全生产的政府监管责任和企业主体责任，完善隐患排查治理和安全防控体系，杜绝重特大事故，防范一般性事故。积极创建国家食品安全示范市和药品放心市，确保人民群众饮食和用药安全。四是继续深化平安创建工作。健全普法宣传教育机制，增强全社会的法治意识。创新立体化的社会治安防控体系，建立重心下移、力量下沉的工作机制，持续开展“打黑除恶”专项行动，严厉打击黄赌毒、暴力恐怖等违法犯罪活动，进一步增强人民群众的安全感。加大依法管理网络力度，确保网络和信息安全。扎实做好民族工作，依法管理宗教事务。

(四)以人为核心加快推进新型城镇化

提升新型城镇化规划水平。树立以公共交通为导向的“一体化”规划理念，统筹考虑城市发展的产业支撑、社会发展、土地利用、基础设施以及人口、资源和环境等因素，系统规划建设城市产业区、商业区、居住区和休闲区，努力做到每一个城市区域都相对功能完备、规模适度、综合品质高、效益最大化。精心编制“一体两翼”重点区域控制性规划，编制好棚户区改造和“美丽乡村”建设规划。指导各县制定修编相关规划，全面启动建制镇规划编制，控制好城镇风貌和强制性指标。

加快“一体两翼”城市建设。围绕建设“山水园林、休闲宜居”城市目标，坚持“秦岭休闲之都、关天产业新城”城市定位，按照“做强一体、提升两翼、组团开发、串珠发展”的思路和“产城一体、板块推进、中疏外扩、成网增绿”的原则，加大“一体两翼”中心城市建设力度，实施城建重点工程87个，其中续建工程36个，新建工程51个，总投资达到174.3亿元，年度投资任务65亿元。集中精力推进“十大工程”，西街旧城改造安置房建成入住，环城北路6月底前建成通车，市文化艺术中心和新建商洛高级中学主体基本建成，商丹城市干线、商洛城市干线完成路基主体工程，商鞅大道及滨江银杏公园建成，市一级客运中心投入运营，环城南线和南秦新区全面启动建设。

强力推进棚户区改造工作。把棚户区改造放在城镇化建设的突出位置，作为完善城市功能、提升城市品位的主要抓手，建立健全中心城市棚户区改造工作管理机制，出台棚户区改造管理暂行办法、调查登记办法以及房屋征收补偿安置标准。按照“政府主导、市场运作，科学规划、分级负责，依法征收、安置先行，配套建设、整片推进”的原则，突出抓好商运区域、黄沙桥片区、通江路东侧等棚户区改造工程，努力把棚户区建成城市发展的新亮点。

积极推进城乡一体化发展。一是加快城乡基础设施建设。加大县城、重点镇和文化旅游名镇建设力度，扎实推进公路交通等基础设施建设，抓好9条干线公路、11条县乡公路、47条通村水泥路建设，新建县乡公路162公里、通村水泥路200公里。持续推进城网农网升级改造，抓好19个电源点建设。二是扎实推进城乡统筹发展。加快镇云及商镇“五位一体”等统筹城乡示范区建设，以提高入住率为重点实施保障性安居工程，完善在建项目综合服务设施，新建项目更加注重选址宜居性，建成项目确保公开公平公正分配，全年新增各类保障性住房4200套，发放住房租赁补贴2100户，完成农村危房改造1.6万户，让群众搬进新居的同时优化城镇布局和功能。鼓励大学毕业生到农村工作，完成86个新型城乡社区建设任务。三是加快农业转移人口市民化。完善农村居民进城落户政策，出台户籍制度改革实施方案，加强就业、社保、教育、医疗等政策衔接，逐步建立覆盖全部常住人口的保障体系。

全力抓好“美丽乡村”建设。把“美丽乡村”建设作为改善农村环境的重要途径，作为促进旅游产业转型升级的重要支撑，作为提升农村群众幸福指数的有力抓手。围绕“环境美、经济强、民风好”的建设要求，按照“重点突破、以点带面”的工作思路，从今年起每年每个区县至少打造2个、全市每年打造15个左右高标准“秦岭美丽乡村”，带动抓好“特色美丽乡村”建设与“全国美丽乡村”创建，形成“普遍提升、明珠镶嵌”的发展局面，推动全市“美丽乡村”建设向纵深发展。

全面加强城市精细化管理。坚持从细微处着眼、以精品意识推进，加快建设安全高效便利的市政公用设施，合理布局公益性菜市场和便民超市，统筹建设电力、通信、给排水、供热、燃气等地下管网。严格落实城市规划建设、公共空间使用有关制度，进一步规范管线布局、公用标识、垃圾分类、停车站点、户外广告等事项。大力倡导科学健康的生产生活方式，扎实做好中心城市创建省级卫生城市复审工作，深入开展园林城市、森林城市、文明城市、生态城镇等创建活动。加大市容环境卫生整治力度，从严查处各类违法建设行为。注重运用信息技术提升城市管理，搞好智慧城市试点，加快智慧城管建设。

(五)以更大的决心和力度改善生态环境

加快推进循环发展和资源综合利用。继续做好国家循环经济示范城市创建工作，以产品上下游衔接和资源综合利用为重点，实施60个循环经济重点工程和尾矿资源综合利用项目，进一步延伸拓展10大循环产业链。加快园区绿色化、生态化、低碳化改造步伐，加大循环园区、循环企业培育力度，形成绿色低碳循环发展新方式。

扎实搞好丹江等流域综合治理。健全断面监测月考核、季评比、定期公布、年终问责制度，深入实施丹江等流域污染防治三年行动计划，系统实施堤防、治污、湿地等生态工程建设，加快完善水源污染事故防范预警应急体系。加强城镇污水管网配套和生活污水处理设施建设，解决好部分污水处理厂运营不正常问题，确保丹江出境水质保持在二类以上。

强力推进治污降霾保卫蓝天行动。全力推进“气化商洛”建设，加快实施“西气东输”三线工程，启动丹凤至商南、商州至洛南、商州至山阳等气化工程，适时开展西安至镇安、柞水气化工程前期工作，努力使全市气化总人口达到12万人。全面推行超净排放、热电联产和脱硝除尘技术，有序拆改城区燃煤锅炉，坚决淘汰黄标车和老旧车，禁止露天焚烧秸秆和垃圾，建立市县联网的大气监控数据中心，确保全市空气质量持续改善。

狠抓资源节约和淘汰落后产能工作。严格落实环境准入、资源环境总量控制、节能减排责任考核等制度，全面做好节地、节水、节能工作。坚持耕地占补平衡，加快划定永久性基本农田，从数量质量两个方面守住耕地红线。坚守水资源开发利用控制、用水效率控制、水功能区限制纳污“三条红线”，实行区域地下水开采总量和地下水位“双控制”。扎实推进工业、建筑、交通和公共机构节能，坚决淘汰高耗能、高污染企业。健全完善资源有偿使用和生态补偿制度，积极开展节能量、碳排放权、排污权、水权交易试点。

深入实施生态修复和农村清洁工程。扎实搞好新一轮退耕还林工作，加快主要河流、公路沿线坡耕地退耕还林，继续抓好中心城市城周绿化和生态绿道建设，统筹实施小流域治理等重点工程，完成造林绿化31万亩，治理水土流失600平方公里。扎实推进工矿废弃地、地质灾害易发区和尾矿库的综合整治，积极防治重金属、危险化学品和危险废物污染。加大农村面源污染治理力度，扩大农村环境连片整治范围，积极推广农村沼气和农业生产废弃物资源化利用。

(六)以更实的举措保障和改善民生

全力打好扶贫攻坚战。坚持“一村一策、一户一法”扶贫方略，以改善发展环境、培育特色产业、增强发展能力为重点，全面深化“双包双促”精准扶贫，投放扶贫贴息贷款3.5亿元，扶持3万贫困户发展产业，抓好115个贫困村整村推进项目。坚持将陕南移民搬迁与新型城镇化建设、美丽乡村建设、“四区”建设相结合，积极推进搬迁安置房、保障房、商品房、棚改房“四房”打通，新建移民搬迁集中安置点90个，搬迁1.6万户。通过综合施策、统筹推进，挖“险根”、除“穷根”，确保8.4万贫困人口实现脱贫。

认真做好就业促进工作。深入开展省级创业型城市创建工作，以高校毕业生、退役军人、城镇困难人员为重点，进一步完善就业创业政策体系，适度放宽创业促就业个人小额贷款额度和贴息年限。新增城镇就业1.5万人，大学毕业生初次就业率保持在85%以上，转移农村劳动力50万人。

加强城乡社会保障工作。积极探索“五保合一”试点，建立社会保险待遇正常增长机制，启动机关事业单位养老保险改革，实现社会保险制度人群全覆盖。按照托底线、保基本、可持续的要求，进一步完善社会救助制度体系，规范城乡低保、农村五保工作，做好救急救难和减灾救灾工作。大力发展社会养老事业，改善社会福利，支持慈善事业，完善农村留守儿童、妇女和老年人关爱服务政策，健全残疾人权益保障制度，抓好农村互助幸福院建设，为各类弱势群体“雪中送炭”。

努力办好人民满意教育。加快实施第二期学前教育三年行动计划，逐步提高学前一年财政补助标准。加快城区中小学校和农村义务教育薄弱学校标准化建设，全面推行教学体制改革和校长、教师交流轮岗制度。继续推进义务教育基本均衡县、“双高双普”和教育强县创建工作，加快普通高中特色化多样化、中职教育规模化实用化发展步伐，支持商洛学院、商洛职业技术学院内涵式发展。重视发展特殊教育，完善困难学生资助体系，鼓励和规范社会力量办学。

提升医疗卫生保障能力。围绕群众“少生疾病、方便看病、看得起病、看得好病”四大目标，加快完善“公共卫生、医疗服务、医疗保障、药品供应”四大体系，突出抓好县级公立医院改革和镇安县医改综合试点工作，健全完善基层首诊、分级诊疗、双向转诊的就医制度。认真落实全科医生制度，有序放开社会力量办医准入。进一步提升大病医疗保障水平，将居民医保政府补助标准同步提高到400元。全面落实计划生育奖扶政策，人口自增率控制在5‰以内。

完善公共文化服务体系。加快基层文化场所建设和文化资源整合，鼓励社会力量参与公共文化服务建设，完成151个行政村(社区)文化活动室设备配送。实施城市数字影院、广播电视无线覆盖、农村电影放映、应急广播、农家书屋等文化惠民工程，支持文化艺术精品创作和群众文化活动，建立政府购买公共文化服务长效机制。加快镇村综合文体中心、全民健身广场建设，广泛开展全民阅读和全民健身活动，不断满足群众日益增长的精神文化需求。大力弘扬社会主义核心价值观，开展“扫黄打非”专项行动，保障文化市场健康有序发展。

四、加快法治政府建设为完成全年任务提供保障

今年是全面推进依法治国的开局之年，我们要结合巩固拓展群众路线教育实践活动成果，深入开展“三严三实”教育，持续打好作风建设这场硬仗，加快建设职能科学、权责法定、执法严明、公开公正、廉洁高效、守法诚信的法治政府。

强化责任意识、严格履职。按照法定职责必须为、法无授权不可为的原则，厘清权力边界，加快推进职能、机构、权限、程序、责任法定化。建立健全权力清单、责任清单和负面清单制度，及时公布行政许可、行政处罚、行政强制、行政征收等事项，坚决杜绝法外设权。依法全面履行政府职能，在简政放权的同时加强事中事后监管，认真履行公共服务、市场监管、社会管理、环境保护等职责，切实解决好与群众生活密切相关的食品安全、市政管理、环境污染等突出问题。

强化法治意识、依法行政。把公众参与、专家论证、风险评估、合法性审查、集体讨论决定，作为重大决策的法定程序。进一步健全政府法律顾问制度，建立重大决策终身追究机制，确保决策制度科学、程序正当、过程公开、责任明确。积极推进综合行政执法体制改革，着力解决权责交叉、多头执法问题。建立健全行政裁量权基准、重大执法决定法制审核、行政执法人员资格管理和持证上岗、执法全过程纪录等制度，坚决查处以言代法、以权压法、徇私枉法等行为。

强化监督意识、规范从政。自觉接受市人大及其常委会的法律监督，认真落实人大决议决定和审议评议意见，自觉接受市政协的民主监督，积极办理议案、建议和批评。加大行政效能监察力度，加快推进审计监督全覆盖，切实加强财政资金分配使用、国有资产监管、政府投资、政府采购、公共资源转让、公共工程建设等重点领域的监督。全面推进政务信息公开工作，高度重视对网络舆情的回应，深入开展公民代表进政府和大学生见习活动。按照守纪律、讲规矩的要求，坚决落实党风廉政建设的主体责任，严肃查处各种违纪违法行为。严格执行党政机关厉行节约反对浪费条例，稳步推进公务用车改革，切实规范公务接待、因公出国出境管理，从严控制“三公”经费支出，腾出更多的财力用于民生事业，让政府过“紧日子”、群众过“好日子”。

强化勤学意识、创新实干。深入学习习近平总书记系列重要讲话，深刻把握新常态和中省的各项新政策，做到既了解宏观、又熟悉微观，练就硬本领、适应新形势，用智慧和勇气积极探索、大胆创新，努力做到谋划发展有点子、破解难题有法子、向上争取有路子，力争更多的工作在全省有亮点、有位次。继续深化“政府执行力提升年”活动，严格落实目标管理、首问负责、按期办结、督查考核和责任追究等制度，大力倡导敢于担当、埋头苦干、务实高效的工作作风。全面推行“三分之一”工作法，市政府班子成员带头用三分之一的时间抓调研谋划、三分之一的时间抓督查落实、三分之一的时间抓外出招商和争取支持，以干部的“辛苦指数”、“勤奋指数”来提升商洛的“发展指数”和群众的“幸福指数”。

各位代表，驻商部队和武警官兵为商洛的改革发展做出了重要贡献，特别是在承担急难险重任务中发挥了生力军作用。我们将紧紧围绕新形势下强军目标，大力支持部队改革和建设，继续深化国防教育，加强国防动员和后备力量建设，扎实开展“双拥共建”活动，不断巩固和发展军政军民团结的大好局面。

各位代表，同心致远、实干兴业。新目标催人奋进，新使命任重道远。让我们紧密团结在以习近平同志为总书记的党中央周围，在省委、省政府和市委的坚强领导下，奋发有为，真抓实干，为加快建设幸福商洛而努力奋斗！
